# The geriatric depression scale and the timed up and go test predict fear of falling in community-dwelling elderly women with type 2 diabetes mellitus: a cross-sectional study

**DOI:** 10.1186/s12877-016-0234-1

**Published:** 2016-03-03

**Authors:** Bruno de Souza Moreira, Daniela Maria da Cruz dos Anjos, Daniele Sirineu Pereira, Rosana Ferreira Sampaio, Leani Souza Máximo Pereira, Rosângela Corrêa Dias, Renata Noce Kirkwood

**Affiliations:** Graduate Program in Rehabilitation Science, Universidade Federal de Minas Gerais, Minas Gerais Belo Horizonte, Brazil; Centro Universitário Estácio, Physical Therapy Course, Minas Gerais Belo Horizonte, Brazil; Nursing School, Physical Therapy Course, Universidade Federal de Alfenas, Minas Gerais Alfenas, Brazil; Department of Physical Therapy, Universidade Federal de Minas Gerais, Minas Gerais Belo Horizonte, Brazil

**Keywords:** Fear of falling, Diabetes mellitus, Elderly, Frailty, Depressive symptoms, Functional mobility, Balance, Gait

## Abstract

**Background:**

Fear of falling is a common and potentially disabling problem among older adults. However, little is known about this condition in older adults with diabetes mellitus. The aims of this study were to investigate the impact of the fear of falling on clinical, functional and gait variables in older women with type 2 diabetes and to identify which variables could predict the fear of falling in this population.

**Methods:**

Ninety-nine community-dwelling older women with type 2 diabetes (aged 65 to 89 years) were stratified in two groups based on their Falls Efficacy Scale-International score. Participants with a score < 23 were assigned to the group without the fear of falling (*n* = 50) and those with a score ≥ 23 were assigned to the group with the fear of falling (*n* = 49). Clinical data included demographics, anthropometrics, number of diseases and medications, physical activity level, fall history, frailty level, cognition, depressive symptoms, fasting glucose level and disease duration. Functional measures included the Timed Up and Go test (TUG), the five times sit-to-stand test (5-STS) and handgrip strength. Gait parameters were obtained using the GAITRite® system.

**Results:**

Participants with a fear of falling were frailer and presented more depressive symptoms and worse performance on the TUG and 5-STS tests compared with those without a fear of falling. The group with the fear of falling also walked with a lower velocity, cadence and step length and increased step time and swing time variability. The multivariate regression analysis showed that the likelihood of having a fear of falling increased 1.34 times (OR 1.34, 95 % CI 1.11–1.61) for a one-point increase in the Geriatric Depression Scale (GDS-15) score and 1.36 times (OR 1.36, 95 % CI 1.07–1.73) for each second of increase in the TUG performance.

**Conclusions:**

The fear of falling in community-dwelling older women with type 2 diabetes mellitus is associated with frailty, depressive symptoms and dynamic balance, functional mobility and gait deficits. Furthermore, both the GDS-15 and the TUG test predict a fear of falling in this population. Therefore, these instruments should be considered during the assessment of diabetic older women with fear of falling.

## Background

Diabetes mellitus is a highly prevalent metabolic disease, particularly in the elderly population [1]. According to the International Diabetes Federation, 134.6 million people (18.6 %) aged between 60 and 79 years were estimated to have diabetes in 2013 [2]. This disease has detrimental effect on multiple organ systems that when combined with the natural aging process and other age-related conditions leads to poorer outcomes compared to those without diabetes [2]. In addition, persons with diabetes tend to have a faster aging process, which puts them at a higher risk of developing frailty at an earlier age [3]. The major cause of frailty is sarcopenia [4], which can be defined as a progressive decline of skeletal muscle mass, strength and quality associated with aging [5]. The factors associated to the development of sarcopenia in persons with diabetes include insulin resistance, glucose toxicity, increased inflammatory cytokine levels, testosterone deficiency and increased fat accumulation [4, 5]. Previous longitudinal study demonstrated that type 2 diabetes is associated with an accelerated loss of leg muscle strength and quality in community-dwelling older adults [6].

The literature reports that elderly population with diabetes is at greater risk for microvascular (retinopathy, nephropathy and neuropathy) and macrovascular (coronary heart disease, cerebrovascular disease and peripheral vascular disease) complications [7]. Diabetes in older adults is also linked to reduced functional status, increased risk of institutionalization and higher mortality [8]. Moreover, several studies have shown that older adults with diabetes fall more often compared to their counterparts without diabetes [9–11]. Reduced muscle strength, poor balance and gait performance alterations have been associated with the history of falls in older adults with diabetes [12]. In this sense, it could be speculated that such deficits may also contribute to the emergence of fear of falling in elderly diabetic patients.

Fear of falling can be defined as a low perceived self-efficacy at avoiding falls during essential, nonhazardous activities of daily living [13]. It is also considered a protective response since it allows the individuals to be more conscious of their surroundings. However, an extreme fear can lead to a restriction of activities, which may generate serious long-term negative effects including physical deconditioning, muscle atrophy, loss of postural control and reduced social participation [14, 15].

Recently, Liu [16], in a cross-sectional study carried out with 445 robust community-dwelling older adults (≥65 years), demonstrated that fear of falling was associated with female gender, poor vision, arthritis, poor performance on the Timed Up and Go (TUG) test, depressive and anxiety symptoms and low self-perceived well-being. Another recent study, using data of 742 participants (≥65 years) from the Research Network Frailty in Brazilian Older People, found that history of falls, use of seven or more medications, hearing impairment, functional dependency in activities of daily living, reduced gait speed, poor self-rated health and depressive symptoms were related to fear of falling [17].

Few studies have investigated fear of falling in population with diabetes [11, 18]. For example, Bruce et al. [18] found that the fear of falling and fear-associated activity restrictions were more common in participants with diabetes than normoglycemic controls. The authors suggested that the increased prevalence of fear of falling in individuals with diabetes could be explained by the excess balance and mobility impairments, obesity, depression and other diabetes-related complications.

In addition, several studies have shown that fearful older adults adopt a more cautious gait pattern, characterized by slower speed, shorter stride length and prolonged double support time [19, 20]. Recently, a meta-analysis provided evidence that the fear of falling is associated with an increase in gait variability in older adults [21]. Additionally, Park et al. [22] demonstrated that a group of older adults with a fear of falling had a significantly worse performance on functional tests - TUG and handgrip strength - compared to those without a fear of falling. To our knowledge, only one study has examined the relationship between the fear of falling and gait data in patients with diabetes, and found that the fear of falling was inversely correlated with gait velocity and stride length (both *r* = −.30) [23]. Furthermore, a recent narrative review pointed out that little is known about fear of falling in older adults with diabetes [5]. Thus, more studies are necessary to better understand the effect of the fear of falling in the functionality of individuals with diabetes, since it is a common and potentially disabling problem among older adults.

Therefore, the objectives of this paper were 1) to investigate the impact of the fear of falling on clinical, functional and gait variables in older women with type 2 diabetes mellitus (DM2) and 2) to determine which variables could predict the fear of falling in this population.

## Methods

### Study design and participants

A cross-sectional observational study was conducted in 99 elderly women with DM2. The participants were recruited from the general community in the city of Belo Horizonte, Brazil. The inclusion criteria were as follows: females aged 65 years or older with DM2, in use of hypoglycemic medication, living independently in the community and able to walk without assistance or walking-devices. The exclusion criteria included cognitive impairment detectable by the Mini-Mental State Examination considering the Brazilian cutoff points based on the degree of education [24], neuropathic symptoms evaluated by the Neuropathy Symptom Score [25] and neurological, orthopedic or rheumatic diseases that could affect physical performance. The present study received approval from the Ethics Committee of the Universidade Federal de Minas Gerais under process number ETIC 0144.0203000-10. All participants signed an informed consent form before participation.

### Data collection

Demographic data, health status and anthropometric measures were collected. Recent blood exam records were consulted regarding the participants’ fasting glucose level. The physical activity level was evaluated by the Brazilian version of the Minnesota Leisure Time Activities Questionnaire (MLTAQ) [26]. This questionnaire is a valid instrument to estimate energy expenditure in leisure time physical activity [27]. The MLTAQ-Brazil presented adequate intra- and inter-rater reliability in community-dwelling elders, with intraclass correlation coefficient (ICC) of .911 and .777, respectively [26]. Depressive symptoms were assessed by the Brazilian version of the Geriatric Depression Scale with 15 items (GDS-15) [28]. The scores range from 0 to 15 points, with a higher score indicating a more depressive state. The Brazilian version of the GDS-15 is a valid tool for the detection of a major depressive episode in older adults [29] and exhibited good level of agreement in test-retest conditions (weighted Kappa = .64) [28]. The frailty level was operationalized by the phenotype created by Fried and co-workers composed of five criteria: unintentional weight loss, self-reported exhaustion, weakness, slow walking speed and low physical activity [30]. In the present study, the cutoff points used to define the presence or absence of frailty in the following criteria - weakness, slow walking speed and low physical activity - were those proposed by Fried et al. [30]. Participants were classified as frail if they presented three or more of the above-mentioned criteria, pre-frail if they presented one or two criteria and non-frail if they presented none of the criteria [30]. The frailty phenotype demonstrated predictive validity for adverse outcomes in older adults, such as falls, disability, hospitalization and death [30].

### Fear of falling assessment

The Brazilian version of the Falls Efficacy Scale-International (FES-I) was used to assess the level of concern about falls when performing 16 activities, from simple in-home activities to more demanding physical and social activities [31]. Each item of the FES-I can be scored from 1 (not at all concerned) to 4 (very concerned) [32]. The total score ranges from 16 to 64. The higher the score, the more fearful the individual is about falling [32]. The fear of falling was also assessed when participants were asked to respond to the yes or no question, “Are you afraid of falling?” The cross-cultural adaptation of the FES-I to the Portuguese language (Brazil) in the community-dwelling elderly population demonstrated adequate internal consistency (Cronbach’s alpha coefficient = .93) and good intra- and inter-rater reliability of the total score (ICC = .84 and .91, respectively) [31].

### Functional tests

The TUG test measured the amount of time the participant required to stand up from an armless chair, walk 3 m, turn 180°, return to the chair and sit down again [33]. This test had concurrent validity of moderate to high when correlated with Berg Balance Scale (*r* = −.81), gait speed (*r* = −.61) and Barthel Index (*r* = −.78) and very high intra- and inter-rater reliability (both ICC = .99) [33]. The five times sit-to-stand (5-STS) test consisted of recording the time needed to rise from a chair and return to the seated position for five repetitions, as fast as possible, with their arms folded across their chest [34]. The validity of this test as a measure of functional strength was supported by the correlation of 5-STS time with knee extension force or torque (*r* = −.48 to −.57) [35]. Regarding the test-retest reliability of the 5-STS, a systematic review summarized the findings of 10 studies and found ICCs ranging from .64 to .96 (adjusted mean = .81) [36]. Handgrip strength in the dominant hand (mean of 3 trials) was obtained using the JAMAR® dynamometer following the guidelines of the American Society of Hand Therapists [37]. The JAMAR® dynamometer presented acceptable concurrent validity with known weights (*r* = .9998) [38] and excellent test-retest reliability over a 12-week interval in apparently healthy community-dwelling elders, with ICC of .954 and .912 for the left and right hands, respectively [39].

### Gait assessment

Gait parameters were measured using a 5.74 m electronic walkway system (GAITRite®, CIR Systems, USA). Gait velocity (cm/s), cadence (steps/min), step length (cm), step time (s), swing time (s), stance time (s) and double support time (s) were collected over six trials at self-selected pace. Gait variability was assessed using the coefficient of variation (CV) of each gait parameter (CV in % = [standard deviation/mean] x 100). Participants started walking 2 m before the carpet and continued 2 m past the carpet to allow for initial acceleration and terminal deceleration. Data from all trials were combined as a single test. The GAITRite® system demonstrated high concurrent validity relative to a gold standard (three-dimensional motional analysis system) [40] and excellent test-retest reliability in older people [41].

### Statistical analysis

The participants were divided in two groups: those with and those without a fear of falling. This distribution was based on a receiver operating characteristic (ROC) curve that was constructed to determine the cutoff point of the FES-I considering the answer to the question “Are you afraid of falling?”, with yes = 1 and no = 0. The optimal cutoff point was the value that maximized the sum of sensitivity and specificity. Categorical variables were presented as percentages and continuous variables as means and standard deviations. Group differences in clinical data, functional tests and gait parameters were determined using the chi-squared test for categorical variables, the independent *t* test for normally distributed continuous variables and the Mann–Whitney test for skewed continuous variables.

A binary logistic regression model was conducted to determine which variables could predict the fear of falling in elderly women with diabetes. Prior to the regression analysis, a factorial analysis with principal component and varimax rotation was performed on a set of 13 gait parameters. This was conducted to reduce the number of gait variables by forming subgroups of new variables, denominated factors, which are uncorrelated and that together explain a large portion of the variance in the data. The goal was to eliminate redundancy between strongly correlated gait parameters, which could lead to incorrect estimates in the regression analysis. The extraction of factors was based on eigenvalues greater than 1.0. The Mann–Whitney test was then conducted with the factor scores to determine which factors were different between the groups with and without the fear of falling.

Variables with a *p*-value less than .20 obtained using the univariate analysis (clinical data, functional tests and the factors extracted) were entered into the forward stepwise logistic regression analysis. The order of entrance of the variables into the model was from the most to the least significant. A significance level of .05 was adopted for permanence of the variables in the final model. Odds ratios (OR) with lower and upper 95 % confidence intervals (95 % CI) were calculated. The existence of multicollinearity among the predictor variables was tested with the Spearman’s correlation test. The adequacy of the multivariate model was evaluated using the Hosmer-Lomeshow goodness-of-fit test, where a non-significant result signifies a good fit. All analyses were performed using the SPSS® software version 20.0, with the level of confidence set at 5 %.

## Results

The FES-I score of the sample ranged from 16 to 64 (24.2 ± 7.7). The area under the ROC curve was .896 (*p* < .05) and the 95%CI was .835 to .956. The cutoff point was set at 23 (sensitivity = 70.1 % and specificity = 93.8 %). Participants with a score < 23 were assigned to the group without the fear of falling (*n* = 50) and those with a score ≥ 23 were assigned to the group with the fear of falling (*n* = 49).

Table 1 shows the results of the univariate analysis. The group with the fear of falling had a significantly higher prevalence of frail individuals, demonstrated more depressive symptoms and exhibited worse performance on the TUG and 5-STS tests compared to the group without the fear of falling. Additionally, participants with a fear of falling walked slower, had lower cadence, took smaller steps and exhibited higher step time and swing time variability than those without a fear of falling. Factorial analysis resulted in a Kaiser-Meyer-Olkin measure, which determines the degree of intercorrelation between variables and the adequacy of the factor analysis, of .783, indicating that the data were adequate for the analysis. Likewise, Bartlett’s test was significant (*p* < .001), indicating enough correlation between the response variables to proceed with the analysis. Table 2 presents the results of the factorial analysis, with two factors accounting for 79.2 % of the variance in gait performance. The first factor accounted for 62.9 % of the variance and was heavily loaded with the variables velocity, cadence, step length, step time, swing time, stance time and double support time. This factor was labeled “spatiotemporal”. The second factor accounted for 16.3 % and was loaded only with gait variability data. We labeled this factor as “variability”. Only Factor 1 was significantly different between the groups (Table 3). The group with fear of falling exhibited lower Factor 1 score (−0.27 ± 1.17) compared to the group without fear of falling (0.26 ± 0.72; *p* = .032), showing that those with fear of falling had worse performance on the “spatiotemporal” domain.

**Table 1:** Clinical, functional and gait variables between the groups with and without a fear of falling

Variables	Fear of falling		*p-*value
	Yes (*n* = 49)	No (*n* = 50)	
*Clinical Data*			
Age (years)	72.6 ± 6.1	71.8 ± 4.7	.758^a^
Body mass index (kg/m^2^)	29.1 ± 4.6	29.4 ± 4.2	.801^b^
Waist-to-hip ratio	1.0 ± 0.1	1.0 ± 0.1	.825^a^
Number of comorbidities	4.2 ± 1.8	3.6 ± 1.5	.069^a^
Number of medications	4.7 ± 2.0	4.5 ± 2.1	.399^a^
MLTAQ (kcal/week)	744.7 ± 905.9	1173.0 ± 1274.9	.051^a^
Fall history			.275^c^
Fallers, %	37.0	26.5	
Non-fallers, %	63.0	73.5	
Frailty level			.016^c,d^
Frail, %	19.1	4.1	
Pre-frail, %	63.8	59.2	
Non-frail, %	17.1	36.7	
MMSE (0–30)	26.6 ± 2.6	26.2 ± 2.8	.963^a^
GDS-15 (0–15)	4.4 ± 2.5	2.7 ± 2.3	.001^a,d^
Fasting glucose (mg/dL)	131.3 ± 39.6	128.1 ± 32.4	.907^a^
Disease duration (years)	9.6 ± 8.5	8.2 ± 8.1	.358^a^
*Functional Tests*			
TUG (s)	11.8 ± 3.7	10.2 ± 1.7	.003^a,d^
5-STS (s)	15.9 ± 5.0	13.8 ± 2.7	.012^a,d^
Handgrip strength (kgf)	19.6 ± 4.4	20.7 ± 4.0	.180^b^
*Gait Parameters*			
Velocity (cm/s)	108.2 ± 21.6	120.1 ± 14.7	.012^a,d^
Cadence (steps/min)	112.4 ± 11.7	117.0 ± 7.6	.023^b,d^
Step length (cm)	57.3 ± 7.9	61.5 ± 5.6	.002^b,d^
Step time (s)	0.54 ± 0.06	0.51 ± 0.03	.111^a^
Swing time (s)	0.42 ± 0.05	0.41 ± 0.03	.283^a^
Stance time (s)	0.66 ± 0.09	0.62 ± 0.05	.083^a^
Double support time (s)	0.24 ± 0.06	0.22 ± 0.03	.197^a^
Velocity CV (%)	4.6 ± 1.9	4.2 ± 2.1	.245^a^
Step length CV (%)	3.5 ± 1.3	3.3 ± 1.1	.352^a^
Step time CV (%)	3.7 ± 2.2	3.1 ± 1.5	.045^a,d^
Swing time CV (%)	4.5 ± 2.4	3.6 ± 1.2	.012^a,d^
Stance time CV (%)	3.6 ± 1.6	3.2 ± 1.8	.073^a^
Double support time CV (%)	8.6 ± 4.4	8.0 ± 3.2	.484^a^

Data are expressed as mean ± standard deviation unless otherwise specified

*MLTAQ* Minnesota Leisure Time Activities Questionnaire, *MMSE* Mini-Mental State Examination, *GDS-15* Geriatric Depression Scale with 15 items, *TUG* Timed Up and Go test, *5-STS* Five times sit-to-stand test, *CV* Coefficient of variation

^a^Mann-Whitney test; ^b^Independent *t* test; ^c^Chi-square test; ^d^Statistical significance

**Table 2:** Rotated component matrix with varimax rotation of the extracted gait factors

Gait parameters	Factor 1 - Spatiotemporal	Factor 2 - Variability
Velocity (cm/s)	.861	
Cadence (steps/min)	.933	
Step length (cm)	.635	
Step time (s)	−.937	
Swing time (s)	−.740	
Stance time (s)	−.956	
Double support time (s)	−.802	
Velocity CV (%)		−.877
Step length CV (%)		−.760
Step time CV (%)		−.877
Swing time CV (%)		−.745
Stance time CV (%)		−.910
Double support time CV (%)		−.762
% of variance explained	62.9	16.3

*CV* Coefficient of variation

**Table 3:** Comparison of the two factors between the groups with and without a fear of falling

Factors	Fear of falling		*p-*value
	Yes (*n* = 49)	No (*n* = 50)	
*Factor 1*			
Spatiotemporal	−0.27 ± 1.17	0.26 ± 0.72	.032^a,b^
*Factor 2*			
Variability	−0.07 ± 0.96	0.07 ± 1.04	.389^a^

Data are expressed as mean ± standard deviation

^a^Mann-Whitney test; ^b^Statistical significance

Of the analyzed variables, eight had a *p*-value less than .20 in the univariate analysis. All correlation coefficients among the predictor variables were less than .50 (data not shown), indicating an absence of multicollinearity. The order of entrance of the variables into the multivariate model was as follows: GDS-15, TUG, 5-STS, frailty level, Factor 1, MLTAQ, number of comorbidities and handgrip strength. Only two variables remained statistically significant at *p* < .05 and composed the final model: GDS-15 and TUG (Table 4). The results showed that the likelihood of having a fear of falling increased 1.34 times for a one-point increase in the GDS-15 score and 1.36 times for each second of increase in the TUG performance. The Hosmer-Lomeshow goodness-of-fit test presented a *p*-value of .453, indicating that the model had a good adjust.

**Table 4:** Final model of the binary logistic regression

Variables	Odds ratio	95 % Confidence interval		*p-*value
		Lower bound	Upper bound	
GDS-15	1.34	1.11	1.61	.003
TUG	1.36	1.07	1.73	.012

*GDS-15* Geriatric Depression Scale with 15 items, *TUG* Timed Up and Go test

## Discussion

The current study revealed that the fear of falling in community-dwelling older women with DM2 without neuropathic symptoms is associated with a higher frailty level, more depressive symptoms, worse performance on functional tests (TUG and 5-STS) as well as alterations in gait parameters, including decreased velocity, cadence and step length and increased variability of step time and swing time. Factorial analysis was also able to identify one domain (spatiotemporal) with significantly worse performance in participants with fear of falling. Additionally, our results demonstrated that the GDS-15 and TUG test predicted the fear of falling in this population.

Older adults with a fear of falling often avoid mobility tasks, such as walking and reaching, which can lead to the deterioration of physical capacities and consequently result in frailty [42]. Prior study demonstrated a relationship between the fear of falling measured with the FES-I and frailty assessed with the Fried et al.’s criteria in a group of high-functioning persons aged 65 to 70 years [43]. The authors observed that vulnerable participants (1 or more frailty criteria) had a reduced fall-related self-efficacy compared with robust participants (no frailty criterion), and concluded that the fear of falling may be an important pathway leading to frailty [43]. As far as we know, our study is the first to show an association between the fear of falling and frailty in older adults with diabetes. We found a significantly higher percentage of frail participants in the group with the fear of falling (19.1 %) compared with the group without the fear of falling (4.1 %). It is important to point out that the frailty phenotype has five components, of which physical activity level and handgrip strength were similar between groups; thus, the presence of the other criteria such as weight loss, exhaustion and gait slowness may have contributed to the greater prevalence of frailty among elderly women with diabetes and fear of falling. This supposition seems to be plausible since we found a lower gait velocity in the elderly women with a fear of falling in comparison with those without a fear of falling when using the GAITRite® system to assess gait parameters.

The GDS-15 is a scale used to screen for depressive disorders in older adults, and a cut-off point of 5/6 (non-case/case) has been recommended [44]. Although both groups had average GDS-15 scores below six points, suggesting absence of depressive disorders, the group with the fear of falling presented a significantly higher GDS-15 score, which means more depressive symptoms. In addition, 33 % and 8 % of the participants with and without a fear of falling, respectively, had depressive disorders (GDS-15 ≥ 6 points; *p* = .002). The link between depressive issues and the fear of falling had been previously demonstrated in various studies [16, 17, 45]. For instance, Malini et al. [17] found that depressive symptoms assessed by the GDS-15 were associated with the fear of falling measured by the FES-I (OR 1.68, 95 % CI 1.07-2.63). Thus, our results support current knowledge and provide new insight regarding the existence of a relationship between symptoms of depression and the fear of falling in elderly individuals with diabetes. Importantly, Munshi et al. [46] found that depressive symptoms and the fear of falling were associated with executive dysfunction in older adults aged 70 years or older with DM2. Executive functions are mental processes that enable us to plan, focus attention, remember instructions and judge multiple tasks successfully [47]. Those are critical functions for performing day-to-day activities. Thus, it is essential to identify elderly with diabetes who are afraid of falling and with symptoms of depression and apply appropriate interventions in order to prevent executive dysfunction and, consequently, functional decline.

The present study also found an association between the fear of falling and decreased performance on functional tests. Of the tests investigated, the TUG and the 5-STS were significantly different between the groups of diabetic elderly women with and without a fear of falling. Similarly, a recent study found that both the TUG and 5-STS tests could discriminate a group of elderly women highly concerned about falls (FES-I score > 20) from an age- and body mass index-matched group of elderly women with low concerns about falls (FES-I score ≤ 20) [48]. The TUG is a quick and practical test designed to evaluate overall performance of the lower limbs, functional mobility and dynamic balance [49]. Recently, Liu [16] and Park et al. [22] also found that older adults with a fear of falling (without a specific disease) spent more time performing the TUG test compared with those without a fear of falling. According to a meta-analysis, elderly individuals aged between 70 and 79 years, which corresponds to the average age of our groups (72.6 and 71.8 years), should perform the TUG test in 9.2 seconds (normal range = 8.2–10.2) [50]. In our study, both groups performed poorly on the TUG test, with one group at the borderline of the normal range (10.2 seconds) and the other with an average time higher than the upper value of the normal range (11.8 seconds), which may be related to diabetes. Previous studies demonstrated that older adults with DM2 have performed poorly on the TUG test than control subjects [9, 51]. The TUG performance of community-dwelling older adults is influenced by a number of factors, such as lower-limb muscle strength, balance, reaction time, vision, health status and cognitive function [52]. Impairments commonly seen in individuals with diabetes may justify the lower performance on the TUG test by the diabetic elderlies. In addition, our results revealed that the presence of fear of falling could have worsened the TUG performance. The group with fear of falling showed an average TUG time of 11.8 seconds, which was 13.6 % higher in comparison with the group without a fear of falling (10.2 seconds). Therefore, fear of falling is an important variable to be considered when assessing the TUG test in older women with DM2.

Traditionally, the 5-STS test is considered a functional measure of muscle strength from the lower limbs [53]. Nonetheless, a previous study showed that older individuals with balance dysfunction had significantly longer 5-STS time than older control individuals, suggesting that the 5-STS test is also a measure of dynamic balance in older adults [54]. A cut-off score of 14.2 seconds was established to identify older adults with balance dysfunction [54]. In the present study, only the group with the fear of falling exhibited an average time of the 5-STS test above the cut-off score cited (15.9 seconds). Thus, our results clearly demonstrate that elderly women with diabetes and fear of falling have a poor functional capacity and present lower-limb muscle strength and dynamic balance deficits, which may negatively spiral toward a loss of confidence, activity avoidance, physical frailty, falls and a loss of independence [14].

Our results also provide original and detailed information on gait performance of elderly women with diabetes who are afraid of falling. In particular, the fear of falling was associated with decreased gait velocity, cadence and step length in addition to reduced spatiotemporal gait performance (Factor 1). Conversely, in a cross-sectional study with 34 patients with diabetes (67.6 ± 9.2 years), Kelly et al. [23] found no difference in gait velocity and stride length across fear of falling levels. These seemingly conflicting findings might be related to differences in characteristics of the samples and the classification of the participants. In their study, eligible subjects were men and women aged 45 years or older with a medical diagnosis of type 1 or type 2 diabetes mellitus. In addition, their participants were classified as having a low, moderate or high concern about falling. Another likely explanation for their non-significant gait comparisons could be the limited statistical power associated with the small sample size.

The average gait velocity of the group with the fear of falling was 108.2 cm/s, which was approximately 10 % slower (difference of 11.9 cm/s) than those without the fear of falling (120.1 cm/s). According to Brach et al. [55], the clinically meaningful change for gait velocity in older adults is 4.15 cm/s for a small change and 10.38 cm/s for a substantial change. Therefore, the difference observed between our groups is substantial and merits consideration since decreased gait velocity was found to be a consistent risk factor for disability, cognitive impairment, institutionalization, falls and mortality in community-dwelling older adults [56].

Gait variability refers to the fluctuation in a gait measure from one step to the next and is thought to represent disruption in intrinsic motor or postural control during walking [57]. There is a general supposition of an inverse relationship between gait variability and gait stability [21]. Thus, high gait variability reflects an inefficient gait control and an unstable gait pattern. In our study, the fear of falling was associated with greater variability in step time and swing time. Previous research demonstrated that greater variability in step time was independently associated with poorer executive function in older adults (72.0 ± 7.0 years), after adjusting for several confounders, including gait velocity [58]. Additionally, a prospective cohort study conducted with 597 participants (80.5 ± 5.4 years) over a mean follow-up period of 20 months, found that increased swing time variability predicted fall risk and injurious falls [59]. These results indicate that an increase in step time variability and/or swing time variability is a marker of health-related adverse outcomes in older populations, including seniors with diabetes as supported by our findings. Although the Factor 2 (variability) was similar between groups, step time and swing time variability did differ between groups. It is important to emphasize that the factor is formed by the variables that present strong correlation with each other and calculated taking into account the weighted average of the items that compose the factor [60]. Therefore, it is possible to observe different results when analyzing individual variables and variable groupings (i.e. factors).

In the present study, there was no association of fall history and age with the fear of falling. Surprisingly, these associations in the literature are inconclusive, which may be partly explained by different population characteristics and methods or concepts used to define the fear of falling. Recently, an updated systematic review whose objectives were to identify additional risk factors for the fear of falling in community-dwelling older adults and analyze those previously mentioned, found a relationship not as robust as expected between the history of falls and the fear of falling [61]. Of the studies identified between 2006 and 2013, 13 presented a significant association between the history of falls and different fear of falling-related constructs, while nine studies exhibited a non-significant association [61]. On the other hand, the relationship between age and fear of falling-related constructs presented an inverse pattern, with eight and 13 studies showing significant and non-significant associations, respectively [61]. Therefore, it appears that fall history and age are not strongly associated with a fear of falling in older individuals.

According to the multivariate analysis, the variables that predicted the fear of falling in our study were the GDS-15 and TUG test. This finding is in agreement with previous studies that also demonstrated that depressive symptoms and TUG performance predicted the fear of falling in older adults [16, 62]. Our results revealed that the increase of one unit in the GDS-15 and TUG test was associated with a 34 % and 36 % higher chance of having a fear of falling, respectively. Depressive symptoms may lead the individual to a less-confident state about his/her physical ability and may become more afraid of falling [45]. On the other hand, the TUG test involves a series of motor tasks requiring integration of the motor, sensory and cognitive systems important for daily activities and independent mobility [33]. Thus, a fear of falling may arise when the individual recognizes deficits in any of these systems. This result is very relevant for clinical practice. Both the GDS-15 and TUG test are simple assessment tools that could help healthcare professionals identify older women with DM2 who are at risk for a fear of falling and, consequently, refer them to appropriate interventions aimed at reducing fear of falling.

This study has some limitations that should be taken into consideration. First, sample selection was based on convenience; thus, the generalization of the results is limited. Second, the cross-sectional design prevents any conclusion about the chronology and the causality of the associations found. Therefore, longitudinal studies are required to clarify the actual causes and consequences of the fear of falling in older individuals with diabetes. Third, diabetes diagnosis was based on self-report. Nevertheless, this has been found to be a reliable method of determining the presence of the disease [63]. In addition, all participants reported the use of hypoglycemic medication, which could have reduced the potential bias related to the diagnosis. Fourth, this study examined the factors associated with the fear of falling solely in women. According to a recent systematic review, the female gender is strongly associated with the fear of falling in community-dwelling older adults [61]. However, it would be interesting to expand our findings to elderly men with DM2. Lastly, although we excluded the participants based on neuropathic symptoms, the peripheral nerve function test was not conducted. Thus, the exact contribution of this factor on the fear of falling in our sample could not be verified. Nevertheless, a prior study found no correlation between the level of peripheral neuropathy and the concern about falling in individuals with diabetes [23].

Despite these limitations, our study has some strong hallmarks. We have established a specific cut-off point to differentiate between those with and without a fear of falling for the community-dwelling elderly population with DM2. This is suitable since using the same cut-off point of other populations with distinct health conditions could lead to different results. Furthermore, our participants were submitted to a comprehensive assessment and various gait parameters were obtained through a gait analysis system widely used in clinical and research settings that allows the register of gait data with great precision. Therefore, several potential variables that could influence the fear of falling in seniors with diabetes were investigated.

## Conclusions

As far as we are aware, this is the first study that examined the impact of the fear of falling on a number of clinical, functional and gait variables in community-dwelling older women with type 2 diabetes mellitus. Our results demonstrated that the fear of falling in this population is related to frailty, depressive symptoms and dynamic balance, functional mobility and gait problems. In addition, multivariate analysis showed that increases in GDS-15 and TUG scores are associated with a greater likelihood of having a fear of falling. Therefore, these instruments should be considered during the evaluation of older women with diabetes and fear of falling.

## Abbreviations

TUG: timed up and go test
*r*: correlation coefficient
DM2: type 2 diabetes mellitus
MLTAQ: minnesota leisure time activities questionnaire
ICC: intraclass correlation coefficient
GDS-15: geriatric depression scale with 15 items
FES-I: falls efficacy scale-international
5-STS: five times sit-to-stand test
CV: coefficient of variation
ROC curve: receiver operating characteristic curve
OR: odds ratio
95 % CI: 95 % confidence interval
